# Staging Language‐Led Neurodegenerative Diseases in a Language‐Based World: The Challenge of the PPA‐Squared Scale

**DOI:** 10.1111/ene.70552

**Published:** 2026-03-27

**Authors:** Salvatore Mazzeo, Massimo Filippi, Maria Salsone

**Affiliations:** ^1^ Vita‐Salute San Raffaele University Milan Italy; ^2^ Neurology Unit, IRCCS Policlinico San Donato San Donato Italy; ^3^ Vita‐Salute San Raffaele University, and Neurology Unit, Neurorehabilitation Unit, and Neurophysiology Service, IRCCS Ospedale San Raffaele Milan Italy

Language is one of the most distinctive capacities of human species. Although other animals are capable of forms of communication, human language has uniquely evolved the ability to generate a theoretically limitless number of messages. This generative property underpins the formation of highly organized societies and sustained advances in explicit knowledge about the world. Language is deeply embedded in everyday life and constitutes the primary communicative medium through which other cognitive functions are expressed and evaluated.

Given this central role, impairment of language produces consequences that extend far beyond communication itself. Language deficits can significantly impact social participation, professional activities, autonomy, and overall quality of life [1].

Humans have also developed heightened sensitivity to the sound and structural patterns of language, such that even minimal deviations in its features (phonology, morphology, lexical retrieval, or pragmatics) are readily detected by native speakers. Unlike many other cognitive functions that are primarily assessed in controlled clinical settings, language is continuously used in everyday life. Consequently, subtle language changes are often noticed by caregivers and family members at very early stages of disease.

From a neurobiological perspective, language relies on an extremely complex and widely distributed neural network, that spans frontal, temporal, and parietal cortices and depends on the integrity of multiple long‐range pathways [2]. This strongly interconnected system makes language particularly vulnerable to disruption by focal lesions, disconnection syndromes, and neurodegenerative processes. In these conditions, even spatially confined pathologic processes may result in severe language impairment, despite minimal involvement of other cognitive domains.

Aphasia represents one of the most severe and disabling consequences of acquired brain injury. In stroke, aphasia affects approximately one third of patients in the acute phase, with a substantial proportion experiencing persistent language deficits [3]. Language impairment also plays a pivotal role in neurodegenerative diseases. Variants of primary progressive aphasia (PPA) frequently constitute the earliest clinical manifestation of two of the most common neurodegenerative disorders: Alzheimer's disease and frontotemporal lobar degeneration [4].

PPA is a syndrome in which language impairment represents the earliest and most prominent clinical manifestation of the underlying neurodegenerative process [5]. The clinical trajectory of PPA is extremely heterogeneous. Patients differ markedly in the rate of progression of linguistic deficits as well as in the emergence and severity of non‐language symptoms. Some individuals show slow progression, maintaining stability in both language and non‐language domains for several years. Others develop severe language impairment (up to total loss of speech) while preserving functional autonomy in daily life, whereas a further subgroup progresses rapidly to profound impairment across multiple cognitive and functional domains [6].

Given this background and the unique characteristics of language, staging systems developed for other dementias are often unreliable when applied to PPA. Language impairment frequently leads to an overestimation of global cognitive decline. For example, the Mini‐Mental State Examination (MMSE), one of the most widely used measures of global cognition, relies heavily on language‐based tasks. As a result, individuals with PPA may obtain low MMSE scores even at early disease stages, despite preserved daily functioning.

Similarly, commonly used dementia staging instruments do not adequately capture language impairment or do not encompass the full spectrum of symptoms associated with PPA. Moreover, as highlighted by Hardy and colleagues [7], most existing staging instruments are primarily based on clinicians' conceptualizations and do not consider the lived experience of patients and caregivers.

To address these limitations, Hardy et al. developed the PPA Progression Planning Aid (PPA‐Squared) [7, 8]. Unlike traditional unidimensional scales, PPA‐Squared adopts a three‐axis model that independently evaluates: (1) verbal communication, (2) non‐verbal cognition and behavior, and (3) personal care and well‐being. Each axis spans a continuum from very mild to profound impairment.

This multidimensional approach offers the important advantage of simultaneously capturing the core aspects of language‐led dementia, assigning equal weight to language, behavior, and functional autonomy. Another major strength of the PPA‐Squared framework lies in its caregiver‐centered perspective. Caregivers play a crucial role in the assessment of dementia and are particularly indispensable in PPA, where patients' language deficits limit their ability to communicate symptoms effectively. Caregivers provide an ecological and longitudinal view of patients' everyday functioning, overcoming the intrinsic limitations of brief clinical evaluations.

The study by Aiello et al. [9] supports the applicability and reliability of PPA‐Squared, providing the first evidence of associations between PPA‐Squared and established cognitive and functional measures. However, as emphasized by Hardy et al., PPA‐Squared should currently be regarded as a proof‐of‐principle prototype that requires further refinement and validation. One open issue concerns the definition of a global stage derived from the three axes. In the study by Aiello et al., the global stage was determined by the most impaired axis. This approach carries the risk of overestimating disease severity, as some patients with PPA may exhibit profound language impairment while retaining near‐normal daily functioning [6]. Indeed, in the Aiello et al. cohort, approximately half of the participants were classified within the two most severe stages.

Another potential limitation of the PPA‐Squared is that, in its current version, it is largely based on data from English‐speaking individuals. Emerging evidence indicates that the clinical manifestation of PPA is shaped by the properties of the language spoken by the patient. For instance, spelling errors (considered a milestone of the very mild stage in the PPA‐Squared for the nonfluent‐PPA variant) are highly salient in opaque orthographies such as English but may be less informative in transparent languages [10]. Conversely, language‐specific symptoms documented in non–Indo‐European languages (such as tonal errors in Chinese or kana and kanji dysgraphia in Japanese), are not currently represented, despite their potential diagnostic and staging relevance. Consequently, application of the PPA‐Squared to languages other than English requires not only translation but also adaptation that accounts for cross‐cultural variability.

Despite these few easily tractable limitations, PPA‐Squared represents an auspicious instrument. Its systematic adaptation across languages could ultimately yield a globally applicable staging framework for individuals with PPA, overcoming current cultural biases and facilitating consistent characterization of disease manifestation and progression. Such a framework would enhance comparability of research findings, improve clinical management across diverse populations, and support the development and evaluation of therapeutic strategies.

## Author Contributions

**Salvatore Mazzeo:** conceptualization, writing – original draft, project administration. **Massimo Filippi:** writing – review and editing, visualization, supervision. **Maria Salsone:** conceptualization, writing – review and editing, visualization, supervision.

## Conflicts of Interest

The authors declare no conflicts of interest.

## Data Availability

Data sharing not applicable to this article as no datasets were generated or analysed during the current study.
